# Spectrum–effect relationship between ultra‐high‐performance liquid chromatography fingerprints and antioxidant activities of *Lophatherum gracile* Brongn

**DOI:** 10.1002/fsn3.2782

**Published:** 2022-02-22

**Authors:** Xiaokang Liu, Yang Wang, Wei Ge, Guangzhi Cai, Yunlong Guo, Jiyu Gong

**Affiliations:** ^1^ 159345 School of Pharmaceutical Sciences Changchun University of Chinese Medicine Changchun China; ^2^ 159345 Jilin Ginseng Academy Changchun University of Chinese Medicine Changchun China

**Keywords:** antioxidant activity, chemical composition, chromatographic fingerprints, *Lophatherum gracile* Brongn, spectrum–effect relationship

## Abstract

*Lophatherum gracile* Brongn. is a medicinal and edible plant resource as well as a natural additive in the functional food market. To better understand its characteristics and efficacy, a method combining chromatographic fingerprints and antioxidant activity was proposed. A total of 21 common peaks were confirmed from liquid chromatography fingerprints and were identified as 14 flavonoids and 7 phenolic acids by ultra‐high‐performance liquid chromatography (UHPLC) coupled with quadrupole Orbitrap mass spectrometry (Q‐Orbitrap/MS). Their antioxidant activities were evaluated by 1,1‐diphenyl‐2‐trinitrophenylhydrazine (DPPH), 2,2'‐diazide‐bis (3‐ethylbenzothiazoline‐6‐sulfonic acid) diammonium salt (ABTS), and ferric reducing antioxidant power (FRAP) assay. The results showed that all of the test samples had moderate to high antioxidant effects, with IC50 values ranging from 5.2 to 16.1 mg/ml and 1.2 to 2.8 mg/ml for DPPH and ABTS assays, and the FeSO_4_ concentrations of 1.84–4.20 mmol/L for the FRAP assay. The spectrum–effect relationship between UHPLC fingerprints and antioxidant activity was investigated through Pearson correlation analysis and Grey relational analysis (GRA) to identify the antioxidant constitutes in *Lophatherum gracile* Brongn. The results showed that 11 compounds were greatly associated with the antioxidant activity with a correlation degree >0.80, which can be used as the quality marker of *Lophatherum gracile* Brongn.

## Graphical abstract

Chromatographic fingerprints of 15 batches of *L. gracile* samples were generated from ultra‐high‐performance liquid chromatography (UHPLC) analysis and the chemical compositions were annotated by UHPLC coupled with quadrupole Orbitrap mass spectrometry (UHPLC‐Q‐Orbitrap/MS). Antioxidant activities were evaluated by 1,1‐diphenyl‐2‐trinitrophenylhydrazine (DPPH), 2,2'‐diazide‐bis (3‐ethylbenzothiazoline‐6‐sulfonic acid) diammonium salt (ABTS), and ferric reducing antioxidant power (FRAP) assay, respectively. The spectrum–effect relationship between UHPLC fingerprints and antioxidant activity was investigated through Pearson correlation analysis and Grey relational analysis (GRA).

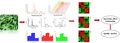

**Novelty impact statement**

The contribution of specific chemical compounds to antioxidant activity was identified by the spectrum–effect relationship‐based approach, which provides us a deeper insight into the joint effect of multiple ingredients of *Lophatherum gracile* Brongn. This study is of great significance for elucidating the pharmacodynamic material basis, screening key quality markers related to medicinal effect, and ensuring the safety and rational application of medicinal herbs.

## 1 INTRODUCTION

Modern pharmacological studies and clinical practices have demonstrated that free radicals produced by normal organ activities will cause chain reactions after exceeding a certain amount in the body, and a series of biological reactions will eventually lead to the occurrence of a variety of diseases, such as cancer, atherosclerosis, hypertension, cataracts, arthritis, and rheumatoid arthritis (Moreno‐Ortega et al., 2020; Raja et al., 2020; Wu et al., 2020). Therefore, antioxidant research has become the forefront of medical research and a hot spot for disease prevention and treatment.

Antioxidants, including both chemically synthesized and naturally occurring antioxidants, have the ability to capture and neutralize free radicals, hence reducing the damage to the organism (Hu et al., 2020). Several chemical synthetic antioxidants like 2,6‐di‐tert‐butyl‐p‐cresol (BHT) and butylated hydroxyanisole (BHA) have been discontinued in some countries due to nephrotoxicity, potential mutagenesis, and teratogenicity concerns (Moretti et al., 2020; Muğlu, 2020). At the same time, the annual growth rate of natural antioxidants’ consumption was 15.6% (Marazza et al., 2012), which indicates that the discovery and development of nontoxic natural antioxidant foods and pharmaceutical products from natural sources has always been a common goal for biology, medicine, and food science research.

*Lophatherum gracile* Brongn. (*L. gracile*) is a perennial herb with dual medicinal and culinary qualities that is extensively used by traditional Chinese medical physicians to treat fever and inflammatory diseases. As a natural additive in the functional food industry, it is a significant ingredient of many herbal teas including “Wong Lo Kat herbal tea” (Ge et al., 2013; Ma et al., 2020). Flavonoids and phenolic acids are plant‐based antioxidants, and many have been identified from *L*. *gracile*, including chlorogenic acid, isoorientin and orientin, isovitexin, cynaroside, and luteolin (Guo et al., 2019), which have antioxidant, antidiabetic, anticardiovascular, anti‐inflammatory, and other properties (Gong et al., 2015; He et al., 2016). For example, they can be used to make facial masks and skin creams, as well as for processing and pickling foods. Besides, antioxidants of *L. gracile* leaves have been approved as a food antioxidant by the Chinese Food Additive Standardization Committee, and have also been applied in the development of food, health care products, medicine, and cosmetics in North America, Asia, and other regions (Kuei‐HungLai et al., 2020).

The leaf of *L*. *gracile* has been officially listed in the Chinese Pharmacopoeia as a crude drug, however, there is no quality control stated except the character identification of the herb. Because few investigations have focused on the quality control of *L*. *gracile*, it is vital to investigate the quality safety and efficacy of *L. gracile*. The fingerprint technique has become a routine technique for the quality control of traditional Chinese herbal medicine (TCHM), but the similarity between chromatograms does not accurately reflect their closeness in drug efficacy (Dabić et al., 2020; Xu et al., 2020; Zhang, Wang, et al., 2019; Zhang, Ding, et al., 2019). Therefore, a spectrum–effect relationship approach has recently been applied to improve the quality assessment of TCHM. A method based on the spectrum–effect relationship was developed for discovering quality markers of the herb *Meconopsis integrifolia* (Huang et al., 2020). Ding and his colleagues established the HPLC fingerprint of different batches of *Panax notoginseng* to determine the promoting blood circulation pharmacodynamic indices of *Panax notoginseng*, and to explore the spectrum–effect relationship between the chemical components and the efficacy of promoting blood circulation (Ding et al., 2021). Liu et al. performed an activity‐calibrated chemical standardization approach for quality assessment of *Salvia miltiorrhiza* Bge (Liu et al., 2017). Those studies indicate that using the spectrum–effect relationship approach to link the “quality” with the potential clinical effects of TCHM is a worthwhile tool.

In this study, the chromatographic fingerprint was established through UHPLC analysis to completely evaluate the quality consistency of 15 batches of *L*. *gracile*, and the common peaks were identified via UHPLC‐Q‐Orbitrap/MS analysis. Three methods, including 1,1‐diphenyl‐2‐trinitrophenylhydrazine (DPPH) method, 2,2'‐diazide‐bis (3‐ethylbenzothiazoline‐6‐sulfonic acid) diammonium salt (ABTS) method, and ferric reducing antioxidant power (FRAP) method, were used for evaluating the antioxidant activity of *L*. *gracile*. Combined with the data of fingerprints and antioxidant activities, the spectrum–effect relationships were established, based on which the antioxidant component in *L*. *gracile* was screened out by Pearson correlation analysis and Grey relational analysis (GRA). This study provides a certain reference basis for the clinical rational use and quality control of *L*. *gracile*.

## 2 MATERIALS AND METHODS

### 2.1 Reagents and materials

Chromatographic‐grade acetonitrile and methanol were obtained from Fisher Scientific. Formic acid in MS grade was provided by Sigma‐Aldrich. Distilled water was produced via a Millipore water purification system (Millipore). All other chemicals were of analytical grade. For standards, luteolin (111,520–201,605), and chlorogenic acid (110,753–202,018) were purchased from NIFDC (National Institutes for Food and Drug Control, Changchun, Jilin, China). Isoorientin (B21528), orientin (B20143), vitexin (B20875), isovitexin (B21544), cynaroside (B20887), DPPH, ABTS, and 2,4,6‐Tris(2‐pyridyl)‐s‐triazine (TPTZ) were obtained from Shanghai Yuanye Bio‐Technology Co., Ltd.

Fifteen batches of *L*. *gracile* herbal materials were collected from their main origin in China and identified by Prof. Jiyu Gong of Changchun University of Chinese Medicine, School of Pharmaceutical Sciences. The information of samples is shown in Table S1.

### 2.2 Sample solution preparation

Fifteen batches of *L*. *gracile* sample solution were prepared. An aliquot of 1.0 g fine powder of each sample was properly weighed and ultrasonically extracted with 20 ml of methanol/water (70/30, v/v) for 20 min at room temperature. The mixture was filtered through a 0.22‐μm membrane filter. The filtrate was transferred into a sample vial for UHPLC and UHPLC‐Q‐Orbitrap/MS analysis. Besides, each powder was accurately weighed and extracted using the same method for the DPPH, ABTS, and FRAP assay.

### 2.3 UHPLC analysis

The UHPLC analysis was performed on a Waters ultra‐high‐performance liquid chromatography system (Waters, Billerica, MA, USA) with diode‐array detection (DAD) detector and a reversed‐phase column (Supelco C_18_ column, 3.0 × 50 mm, 2.7 μm; Sigma‐Aldrich) maintained at 30°C. The mobile phase consisted of eluent A (0.05% formic acid aqueous solution) and eluent B (acetonitrile) at a flow rate of 0.4 ml/min with the following gradient program: 0–5 min, 5% (B); 5–12 min, 5%–10% (B); 12–15 min, 10%–15% (B); 15–20 min, 15%–42% (B); 20–25 min, 5% (B). The injection volume was 5 μl.

### 2.4 UHPLC‐Q‐Orbitrap/MS analysis

Chromatographic separation was conducted using the same method as UHPLC analysis. And the elute was introduced into a Q‐Orbitrap‐MS (Thermo Fisher Scientific) equipped with an electrospray ionization source under the negative ion mode. The parameters of the ion source were set to 40 Arb for sheath gas flow, 10 Arb for auxiliary (aux) gas flow, and 1 Arb for sweep gas flow. The S‐Lens radio frequency (RF) was 55%. The capillary voltage was set to −3.5 kV with a capillary temperature of 350°C. Full MS data were acquired at the centroid mode from m/z 150 to 2000 Da, using the resolution of 70,000, with the automatic gain control (AGC) target of 1 × 10^6^ and maximum injection time (IT) of 100 ms. The tandem mass spectrum was obtained in Full‐MS/ddMS2 mode using the following settings: 17,000 for resolution, 1 × 10^5^ for automatic gain control (AGC) of the target, 50 ms for maximum IT, 5 for Loop count, 5 for TopN, 4.0 m/z for Isolation window, and 25, 35, 55 for stepped normalized collision energy (NCE).

### 2.5 Antioxidant activities

#### 2.5.1 DPPH assay

All *L*. *gracile* samples were tested for DPPH radical scavenging activity according to the previous report (Guo et al., 2019) with minor modifications. In brief, an aliquot (100 μl) of each sample was added to 100 μl of DPPH solution to initiate the reaction, and 70% methanol was used as the blank solution. After 30 min of incubation in the dark at room temperature, the absorbance was measured at 517 nm. Each measurement was performed in triplicate. The DPPH quenched rate was calculated according to the following equation:

$$\text{DPPH}\;\text{activity}\left(\%\right)=\left(1‐A_{\text{sample}}/A_{\text{blank}}\right)*100$$

Where A*_blank_* and A*_sample_* represent the absorbances of the blank sample and test sample, respectively.

#### 2.5.2 ABTS assay

The ABTS radical scavenging activity of each sample was determined using Mohammad Noshad's technique (Noshad et al., 2021) with slight modifications. The same volumes of 7.4 mM ABTS solution and 2.60 mM K_2_S_2_O_8_ were mixed and kept at room temperature for 12 h under dark conditions to prepare the stock solution. Before use, the ABTS working solution was obtained by adding 70% methanol to the stock solution until the absorbance reached 0.70 at 734 nm. Thereafter, 50 μl of each sample solution was mixed with the 800 μl of ABTS working solution and was kept at ambient temperature for 10 min. After that, the absorbance at 734 nm against the blank sample (70% methanol) was measured and recorded. All measurements were done in triplicate. The ABTS activity of each sample was calculated according to the following formula:

$$\text{ABTS}\;\text{activity}\left(\%\right)=\left(1‐A_{\text{sample}}/A_{\text{blank}}\right)*100$$

#### 2.5.3 FRAP assay

The ability to reduce ferric ions was measured based on previous articles (Krivokapic et al., 2021; Tadic et al., 2021) with minor revisions. The stock solutions included 300 mM acetate buffer, pH 3.6, 10 mM 2,4,6‐Tris(2‐pyridyl)‐s‐triazine (TPTZ) solution in 40 mM HCl and 20 mM FeCl_3_.6H_2_O solution. The working solution was prepared by mixing 25 ml of acetate buffer, 2.5 ml of TPTZ solution, and 2.5 ml of FeCl_3_.6H_2_O solution and then warming at 37°C before use. Each sample solution (50 μl) was used to react with 200 μl of FRAP reagent at room temperature for 10 min and the absorbance of the reaction mixture was measured at 593 nm. The 100 mmol/L FeSO_4_ was used as the standard solution for calibration. All tests were carried out in triplicate. The total antioxidant capacity of reducing ferric ions of each sample was expressed by the amount of substance produced which represents that Fe^3+^‐TPTZ is reduced to Fe^2+^‐TPTZ.

### 2.6 Data processing and multivariate statistical analysis

The raw data from UHPLC were converted into.AIA format file and then imported into the Similarity Evaluation System for Chromatographic Fingerprint of Traditional Chinese Medicine software (version 2012A) for peak alignment and similarity analysis. The reference fingerprint was generated by the system using the Median method from the general comparison of the chromatograms of 15 *L*. *gracile* extracts and the similarity values between the reference fingerprint and the chromatograms of different sample extracts were calculated by the cosine of vectorial angle method using this software.

The Pearson correlation analysis and GRA were performed using the Hiplot web tool and SPSSAU (Version 21.0, online application software), respectively, to find the spectrum–effect relationship between common peaks and antioxidant activities and further to characterize the quality of *L*. *gracile*.

## 3 RESULTS AND DISCUSSION

### 3.1 Establishment of the UHPLC fingerprint of *L*. *gracile* sample

**FIGURE 1:**
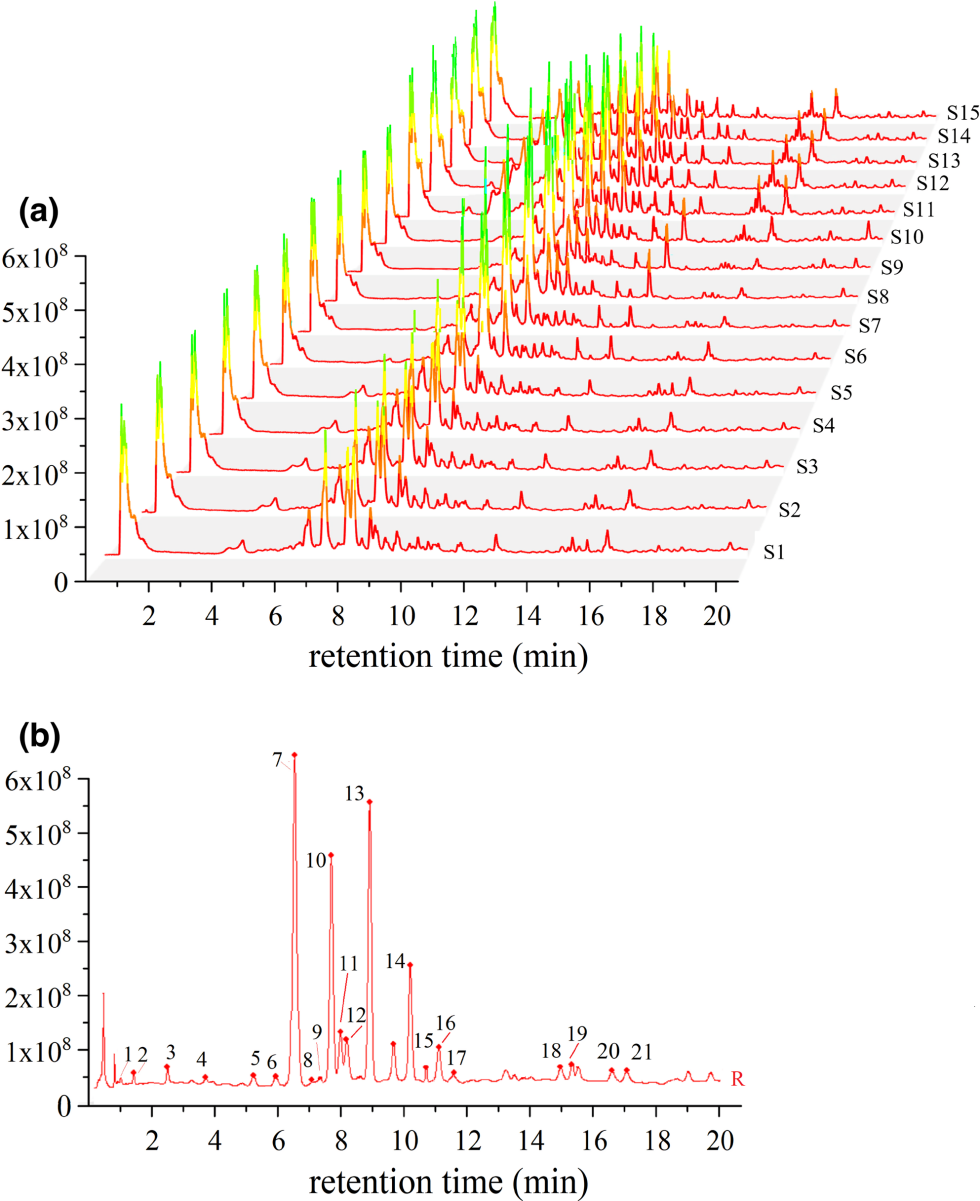
Ultra‐high‐performance liquid chromatography (UHPLC) fingerprints of 15 batches of *L*. *gracile* (a) and reference fingerprint chromatogram (b)

Figure 1 shows the UHPLC fingerprints and the reference fingerprint of *L*. *gracile* extracts from 15 batches of samples. Under the current chromatographic conditions, a total of 21 peaks have peak areas exceeding 90% of the total area, and their resolution in the chromatogram is good, thus they are regarded as common characteristic peaks. The similarity evaluation was calculated by using S1 as the reference chromatogram after a multipoint calibration. The similarity values of 15 introduced *L*. *gracile* were in the range of 0.903–0.989, indicating that the samples from different batches had similar chemical compositions and that the origin is not the key factor influencing sample quality diversity.

### 3.2 Identification of chemical components from *L. gracile*

**FIGURE 2:**
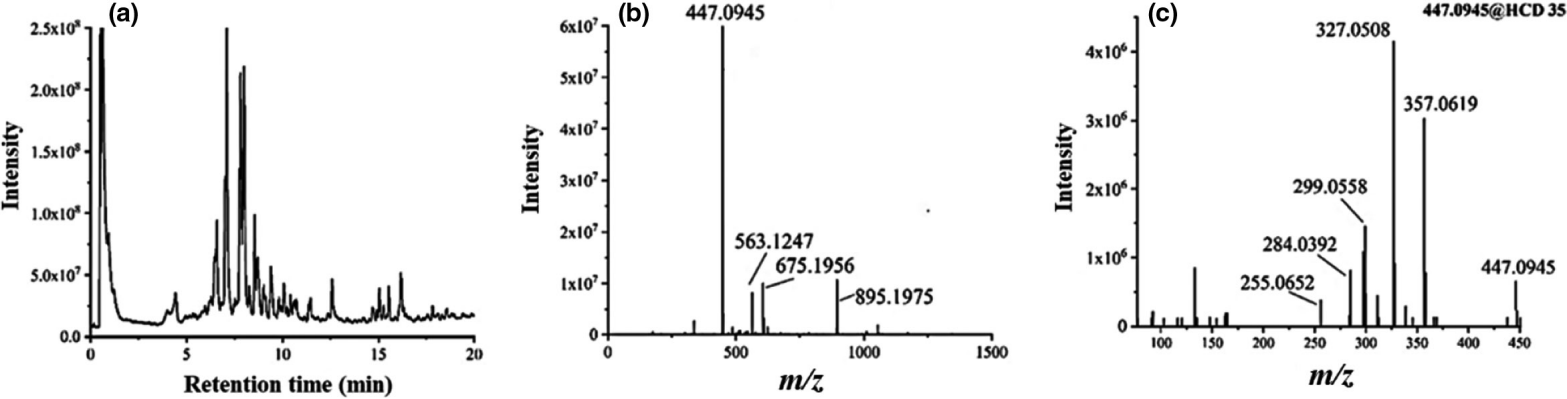
Base peak intensity (BPI) chromatograms of *L*. *gracile* (a), full‐scan mass spectrum (b), and tandem mass spectrum (c) of isoorientin

UHPLC‐Q‐Orbitrap/MS was used to profile the chemical composition of *L*. *gracile*. The representative base peak intensity (BPI) chromatograms of the sample are shown in Figure 2a. By comparing the MS and MS/MS information of detected compounds with the database, literature records, and standard references, a total of 21 compounds were tentatively identified, and the annotation information is listed in Table 1.

**TABLE 1:** Identification of 21 common peaks in *L*. *gracile* by ultra‐high‐performance liquid chromatography coupled with quadrupole Orbitrap mass spectrometry (UHPLC‐Q‐Orbitrap/MS)

Peak No.	RT (min)	Identity	Formula	Detected *m/z*	Mass error (ppm)	Fragment ion	References
P1	1.28	Neochlorogenic acid	C_16_H_18_O_9_	353.0894	4.5	191.0555, 179.0336, 173.0442, 135.0435	(Guo et al., 2019; Tang et al., 2015)
P2	1.31	Chlorogenic acid^a^	C_16_H_18_O_9_	353.0889	3.1	191.0552, 179.0339, 173.0441, 163.0386, 135.0437	(Gong et al., 2015; Ma et al., 2020)
P3	2.17	Cryptochlorogenic acid	C_16_H_18_O_9_	353.0885	2.0	191.0551, 179.0337, 173.0451, 135.0438	(Guo et al., 2019)
P4	3.42	5‐O‐Coumaroylquinic acid	C_16_H_18_O_8_	337.0945	4.7	191.0550, 163.0389, 119.0488	(Tang et al., 2015)
P5	4.65	Feruloylquinic acid	C_17_H_20_O_9_	367.1050	4.1	193.0496, 173.0449, 149.0594, 134.0360	(Gong et al., 2015; Tang et al., 2015)
P6	6.13	3‐O‐Coumaroylquinic acid	C_16_H_18_O_8_	337.0939	3.0	191.0551, 173.0444, 163.0387, 119.0488	(He et al., 2016; Tang et al., 2015)
P7	6.29	4‐O‐Coumaroylquinic acid	C_16_H_18_O_8_	337.094	3.3	173.0444, 163.0389	(Gong et al., 2015; Ma et al., 2020)
P8	6.92	Luteolin‐6‐C‐β‐D‐galactopyranosiduronic acid (1 → 2)‐β‐D‐glucopyranoside	C_27_H_28_O_17_	623.126	1.0	543.9896, 409.1641, 340.5211	(Guo et al., 2019; Tang et al., 2015)
P9	7.48	Luteolin‐7‐O‐β‐D‐glucopyranosyl‐6‐C‐α‐L‐arabinopyranoside	C_26_H_28_O_15_	579.1305	−8.6	539.4812, 465.9688, 369.0574	(Gong et al., 2015; Tang et al., 2015)
P10	7.71	Isoorientin^a^	C_21_H_20_O_11_	447.0945	2.7	357.0616, 327.0508, 299.0558, 284.0329, 255.0625	(He et al., 2016; Ma et al., 2020).
P11	8.02	Swertiajaponin	C_22_H_22_O_11_	461.1111	4.8	341.0661, 313.0352, 298.0479, 285.0397	(Guo et al., 2019; Tang et al., 2015)
P12	8.49	Luteolin‐6‐C‐β‐D‐galactopyranosiduronic acid (1 → 2)‐α‐L‐arabinopyranoside	C_26_H_26_O_16_	593.1164	2.7	417.0809, 399.0727, 357.0607, 327.0506	(Guo et al., 2019; Tang et al., 2015)
P13	9.11	Orientin^a^	C_21_H_20_O_11_	447.0943	2.2	357.0603, 327.0504, 299.0544, 285.0397	(He et al., 2016; Ma et al., 2020).
P14	10.41	Cynaroside^a^	C_21_H_20_O_11_	447.0923	−2.2	357.0612, 327.0507, 298.0465, 285.0393	(He et al., 2016; Tang et al., 2015)
P15	10.71	Tricin−7‐O‐β‐D‐glucoside	C_23_H_24_O_12_	491.1186	−1.8	491.1190, 466.5033, 414.5631, 313.0347	(Guo et al., 2019; Tang et al., 2015)
P16	11.08	Vitexin^a^	C_21_H_20_O_10_	431.0976	−1.9	341.0664, 311.0562, 298.0482	(He et al., 2016; Ma et al., 2020)
P17	11.45	Isovitexin^a^	C_21_H_20_O_10_	431.0989	1.2	341.0664, 311.0562, 298.0482	(Ma et al., 2020)
P18	15.26	Swertisin	C_22_H_22_O10	445.1167	6.1	385.4311, 355.2131, 325.3123, 297.0342, 285.0397	(Guo et al., 2019; He et al., 2016)
P19	15.44	Afzelin	C_21_H_20_O_10_	431.0976	−1.9	413.1742, 401.9022, 387.2316, 285.4217	(Guo et al., 2019)
P20	16.25	Luteolin^a^	C_15_H_10_O_6_	285.0416	3.9	285.1703, 179.4183, 162.5418	(Guo et al., 2019; Tang et al., 2015)
P21	16.95	Apigenin	C_15_H_15_O_5_	269.2406	5.6	225.1477, 176.4940, 116.4693	(Gong et al., 2015; Guo et al., 2019)

^a^ These compounds were confirmed by standard references.

The detailed annotation procedures were demonstrated by taking the ion of isoorientin at RT 7.71 min_*m/z* 447.0945 as an example. As shown in Figure 2b, the base peak at *m/z* 447.0945 is the deprotonated molecule of isoorientin. The tandem MS spectrum is shown in Figure 2c, and the ions at *m/z* 367.0619 and 327.0508 were generated by the loss of C_3_H_6_O_3_ and C_4_H_8_O_4_ groups. The ions at *m/z* 299.0558 and 284.0329 were formed from the loss of CO and C_2_H_3_O group, respectively, based on the ion at *m/z* 327.0508. And the loss of CO_2_ from ion at *m/z* 299.0558 generates the ion of 255.0652. The proposed fragmentation pathway is shown in Figure S1. Collectively, this ion at RT 7.71 min_*m/z* 447.0945 was assigned to isoorientin.

### 3.3 DPPH free radical scavenging activity

**FIGURE 3:**
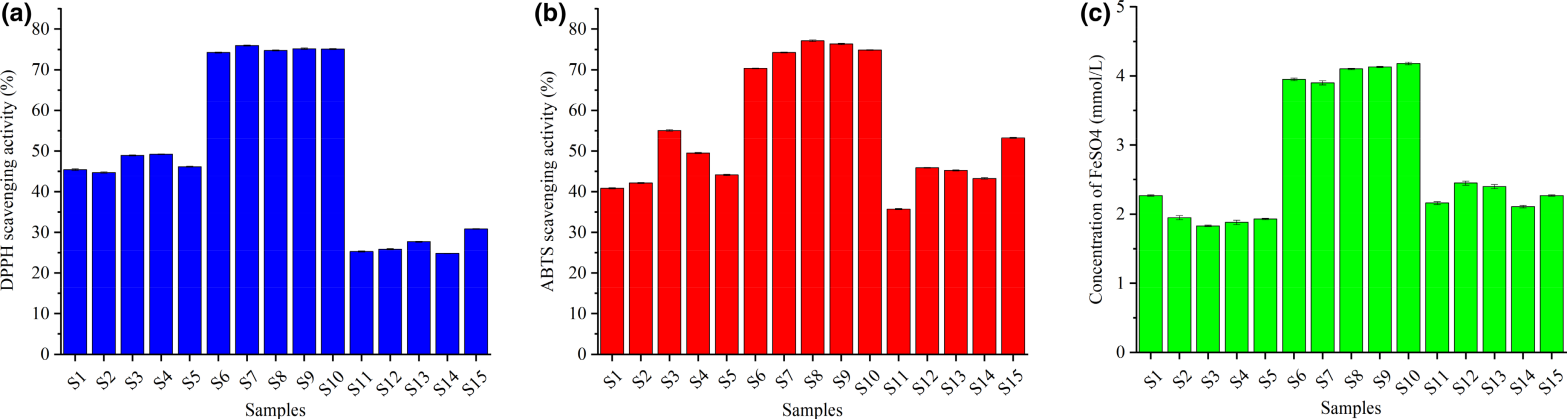
The results of 1,1‐diphenyl‐2‐trinitrophenylhydrazine (DPPH) (a), 2,2'‐diazide‐bis (3‐ethylbenzothiazoline‐6‐sulfonic acid) diammonium salt (ABTS) (b), and ferric reducing antioxidant power (FRAP) (c) antioxidant assay

The studies using varying concentrations (5–20 mg/ml) of *L*. *gracile* extracts were first conducted, and the result is shown in Figure S2a. The antioxidant activity of *L*. *gracile* was evaluated using the half‐maximal inhibitory concentration (IC_50_) values. The better the antioxidant activity, the lower the IC50 levels. In the case of samples from Dazhou city, our results exhibited an outstanding DPPH scavenging activity with IC_50_ ranging from 5.2 to 5.4 mg/ml, which is lower than those of the samples from Anhui province (7.9–8.8 mg/ml) and Chengdu city (12.7–16.1 mg/ml). The scavenging activity of extracts from *L*. *gracile* samples against the stable radical DPPH was evaluated at a concentration of 12.5 mg/ml and the result is illustrated in Figure 3a. The samples collected from Dazhou city (S6–S10) showed a high scavenging activity (>75%). The scavenging activity of samples collected from Anhui province exhibited the scavenging activity of 40%–50%, which was higher than the sample harvest from Chengdu city ranging from 25% to 30%.

### 3.4 ABTS free radical scavenging ability

The IC_50_ value of these samples was investigated using varying concentrations (1–10 mg/ml). As observed with the ABTS assay, lower IC_50_ values (1.2–1.5 mg/ml) were obtained from the sample S6–S10 that was collected from Dazhou city (Figure S2b). The ABTS radical scavenging activity is based on the reduction of ABTS^+^ to ABTS through a hydrogen atom transfer mechanism, in the presence of antioxidant agents (Zhao et al., 2017). The scavenging activity of various extracts (10 mg/ml) of *L*. *gracile* is presented in Figure 3b. Better scavenging activity is obtained from the samples collected from Dazhou city (>70%), which is in agreement with the results obtained by the DPPH assay. A similar scavenging activity was observed for the sample harvest from Anhui province and Chengdu city, ranging from 35% to 70%.

### 3.5 Ferric ion reducing antioxidant power (FRAP) assay

The FRAP assay measures the substance's ability to reduce Fe^3+^ to Fe^2+^ through electron transfer. According to the above procedure, the calibration curves were constructed by plotting the absorbance versus the concentrations of FeSO_4_ solution. Satisfactory calibration curves of the FeSO_4_ solution were obtained, *A = 0.0392C + 0.0622*, with a high correlation coefficient value (*r^2^* *= 0.992*) showing good linearity at a relatively wide range of concentrations (1–100 mmol/L). The ferric reducing activities of the various extracts (20 mg/ml) *L*. *gracile* samples were determined using the FRAP values. As shown in Figure 3c, the total antioxidant activity of the 15 batches of *L*. *gracile* showed significant differences, and the FeSO_4_ concentrations of the 5 samples from Anhui province (1.84–2.29 mmol/L) were similar to those of the samples from Chengdu city (2.13–2.93 mmol/L) but lower than those of the samples from Dazhou city (3.94–4.20 mmol/L).

Due to different growing habitats and conditions, the antioxidant activities of *L*. *gracile* growth in different regions differed from each other. Thus, the information regarding 15 batches of *L*. *gracile* was sufficient for the spectrum–effect analysis.

### 3.6 Studies on the spectrum–effect relationship analysis

In the present study, the spectrum–effect relationships between chromatographic fingerprint peaks and antioxidant activity of *L*. *gracile* samples were investigated by Pearson correlation analysis and GRA.

#### 3.6.1 Pearson correlation analysis

**FIGURE 4:**
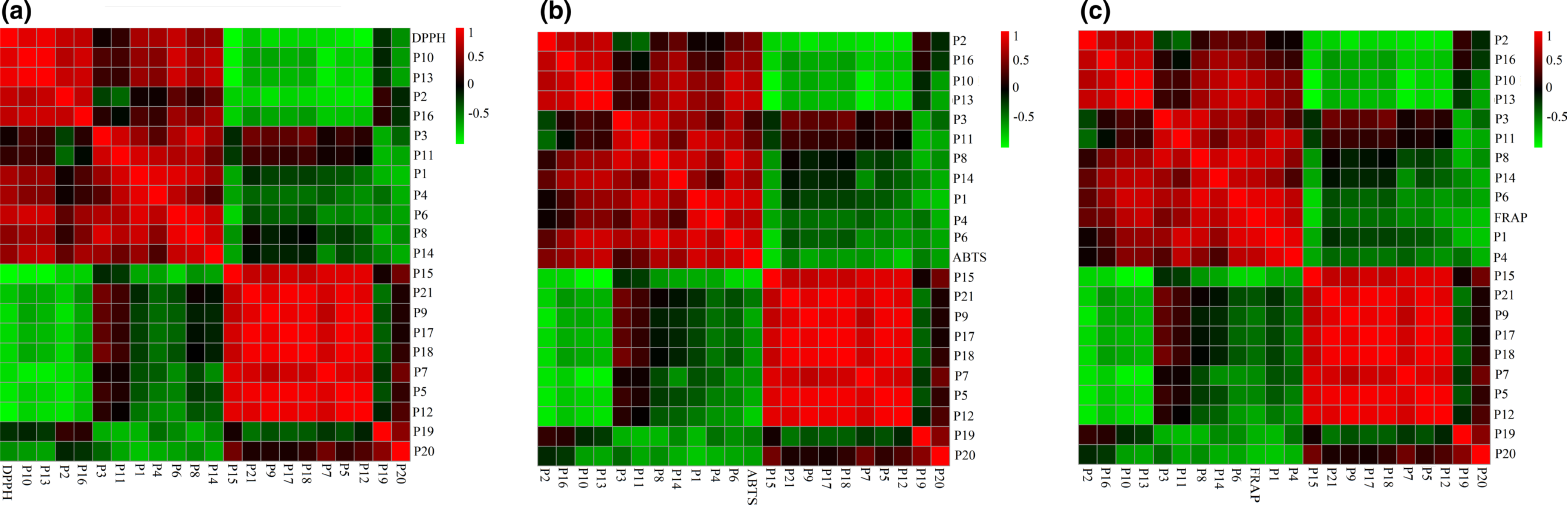
Heatmap analysis of Pearson correlation of 21 common peaks (P1–P21) areas and antioxidant activities; scavenging value of 1,1‐diphenyl‐2‐trinitrophenylhydrazine (DPPH) (a), 2,2'‐diazide‐bis (3‐ethylbenzothiazoline‐6‐sulfonic acid) diammonium salt (ABTS) (b), and concentration of FeSO_4_ for ferric reducing antioxidant power (FRAP) (c) assay. Red represents positive correlation and green indicates negative correlation

Pearson correlation analysis is widely used to determine which factors have the greatest impact on the result variables and to optimize the correlations between the two sets of variables. In this study, three Pearson correlation models that were initially established by taking the areas of 21 common peaks in the UHPLC fingerprints were recognized as one set of variables, and the mean values of antioxidant activities from 15 samples, including the free radical scavenging activity values of DPPH and ABTS assay, and the FeSO_4_ concentration of FRAP assay as the other set, respectively. A positive correlation occurs when the correlation coefficient (*r*) is between +0.5 and +1.0, whereas a negative correlation comes about when the correlation coefficient (*r*) is between −0.5 and −1.0. As shown in Figure 4, the peaks P10, P13, P2, P16, P1, P4, P3, P11, P14, P6, and P8 from *L*. *gracile* were positively correlated with the scavenging activity values of DPPH and ABTS, and the FeSO_4_ concentration of FRAP assay, which indicated that these compounds were the primary bioactive components of *L*. *gracile* involved in the antioxidant effect.

#### 3.6.2 Grey relational analysis

The GRA was also used to investigate the relationship between the antioxidant effects and 21 common peaks found in *L*. *gracile* samples, and further to identify the primary active compounds responsible for these effects. After data standardization, the correlation coefficient was calculated. The correlation coefficient was utilized to determine the association between chemical components and antioxidant capacity, the greater the correlation coefficient, the stronger the association between the chemical composition and the antioxidant capacity. As listed in Table 2, peaks with a correlation degree >0.80 were ranked in the following sequence: P13>P6>P10>P16>P2>P1>P4>P8>P11>P3>P14>P19>P20 for DPPH assay, P13>P1>P16>P10>P4>P3>P14>P8>P2>P11>P21>P6>P18>P20>P15 for ABTS assay, and P13>P10>P11>P8>P1>P2>P16>P4>P14>P6>P3 for FRAP assay, which was largely consistent with the results of our Pearson correlation analysis shown above, further confirming these components in *L*. *gracile* were greatly associated with antioxidant bioactivity.

**TABLE 2:** Grey relational coefficient (GRA) between fingerprints and antioxidant activities of *L*. *gracile*

Peak No.	Compound	DPPH assay		ABTS assay		FRAP assay	
		Correlation coefficient	Rank	Correlation coefficient	Rank	Correlation coefficient	Rank
P1	Neochlorogenic acid	0.817	6	0.894	2	0.863	5
P2	Chlorogenic acid	0.826	5	0.818	9	0.856	6
P3	Cryptochlorogenic acid	0.805	10	0.826	6	0.801	11
P4	5‐O‐Coumaroylquinic acid	0.814	7	0.833	5	0.844	8
P5	Feruloylquinic acid	0.704	18	0.671	18	0.787	12
P6	3‐O‐Coumaroylquinic acid	0.911	2	0.805	12	0.805	10
P7	4‐O‐Coumaroylquinic acid	0.694	19	0.607	20	0.756	14
P8	Luteolin‐6‐C‐β‐D‐galactopyranosiduronic acid (1 → 2)‐β‐D‐glucopyranoside	0.811	8	0.819	8	0.8	4
P9	Luteolin‐7‐O‐β‐D‐glucopyranosyl‐6‐C‐α‐L‐arabinopyranoside	0.604	21	0.516	21	0.753	16
P10	Isoorientin	0.84	3	0.864	4	0.918	2
P11	Swertiajaponin	0.808	9	0.813	10	0.909	3
P12	Luteolin‐6‐C‐β‐D‐galactopyranosiduronic acid (1 → 2)‐α‐L‐arabinopyranoside	0.767	17	0.622	19	0.656	20
P13	Orientin	0.935	1	0.942	1	0.938	1
P14	Cynaroside	0.803	11	0.825	7	0.826	9
P15	Tricin‐7‐O‐β‐D‐glucoside	0.796	14	0.8	15	0.694	19
P16	Vitexin	0.831	4	0.869	3	0.854	7
P17	Isovitexin	0.795	15	0.725	16	0.731	17
P18	Swertisin	0.611	20	0.802	13	0.776	13
P19	Afzelin	0.801	13	0.723	17	0.654	21
P20	Luteolin	0.803	12	0.801	14	0.704	18
P21	Apigenin	0.793	16	0.808	11	0.755	15

By combining the results of Pearson correlation analysis and GRA, excluding the compounds that were negatively related to the antioxidant activity and had low correlation degree in GRA, 11 compounds, including P1 (Neochlorogenic acid), P2 (Chlorogenic acid), P3 (Cryptochlorogenic acid), P4 (5‐O‐Coumaroylquinic acid), P6 (3‐O‐Coumaroylquinic acid), P8 (Luteolin‐6‐C‐β‐D‐galactopyranosiduronic acid (1 → 2)‐β‐D‐glucopyranoside), P10 (Isoorientin), P11 (Swertiajaponin), P13 (Orientin), P14 (Cynaroside), and P16 (Vitexin), were considered to contribute the most to the antioxidant capacity of *L*. *gracile* and could be selected as quality markers for its quality control.

Orientin, vitexin, isovitexin, and homoorientin are four representative flavone C‐glucosides in *L*. *gracile*, which have been considered as the main antioxidants in previous reports (Gong et al., 2015; Guo et al., 2019; Ma et al., 2020). Accordingly, our study showed that isoorientin, orientin, and vitexin had strong scavenging effects on the DPPH radical and hydroxyl radical with a higher correlation. Besides, cynaroside, swertiajaponin, and luteolin‐7‐O‐β‐D‐glucopyranosyl‐6‐C‐α‐L‐arabinopyranoside also had a high correlation, which agreed with the previous studies (Ge et al., 2013; He et al., 2016; Ma et al., 2020). In addition to the flavones, phenolic acid compounds in *L*. *gracile* include cryptochlorogenic acid, chlorogenic acid, neochlorogenic acid, 5‐O‐coumaroylquinic acid, and 3‐O‐coumaroylquinic acid, which also exhibited good antioxidant activities (Gong et al., 2015; Ma et al., 2020; Tang et al., 2015). The above reports demonstrated the applicability of using the selected 11 compounds to evaluate the antioxidant effect of *L*. *gracile*.

Since ancient times, *L*. *gracile* has been utilized as an antioxidant to prevent food deterioration. The main active ingredients have been identified as flavonoids, polysaccharides, and phenolic acids. The presence of hydroxyl groups in the structure of the phenolic compounds of *L*. *gracile* substances is often linked to their antioxidant activity (Gong et al., 2015).

In this study, the antioxidant effects of *L*. *gracile* were investigated. The results showed that all of the test samples had moderate to high DPPH and ABTS scavenging activities, with IC_50_ values ranging from 5.2 to 16.1 mg/ml and 1.2 to 2.8 mg/ml, respectively (Figure S2). The antioxidant activity of different batches of *L*. *gracile* samples was different, which might be related to the variance of bioactive components in samples. Therefore, we further investigated the potential antioxidant compounds from *L*. *gracile*.

Discovering bioactive molecules from herbal medicine is difficult, due to its property of multiple chemical components and multiple targets. The most prevalent methods concentrate on compound separation and single component activity, which are time‐consuming and fail to reveal the complex roles of numerous components in herbal medicines (Liu et al., 2017). The spectrum–effect relationship analysis is an innovative and dependable technology that integrates chromatographic fingerprints with pharmacological effects by chemometrics, which could be used to investigate the correlations between bioactive components and efficacy, as well as to identify the major bioactive elements in herbal medicines (Huang et al., 2020; Zhang, Ding, et al., 2019).

The antioxidant properties of *L*. *gracile*, according to the data, were dominated by several compounds rather than a single substance. The results of Pearson correlation analysis and GRA showed that Peaks P1, P2, P3, P4, P6, P8, P10, P11, P13, P14, and P16 were positively associated with antioxidant activities and had a high correlation degree of greater than 0.80, which suggested that these 11 compounds played important roles in the antioxidant activity of *L*. *gracile*, and could be used as supplements in medicinal foods and pharmaceuticals, as well as to ensure the safety and rational application of *L*. *gracile*.

The characterization and quantification of all active chemical ingredients of TCHM face substantial hurdles due to the variety and complexity of the chemical composition of TCHM. At present, the application of chemical fingerprints to evaluate the quality of TCHM has been widely accepted and used in many countries. However, the assessment of pharmacodynamic effects will be overlooked when only analyzing the type and content of its chemical components to evaluate the quality of TCHM. The investigation of spectrum–effect relationships, which combine the chemical composition of TCHM with its pharmacodynamic activity, allows for a more thorough assessment of drug quality.

## 4 CONCLUSION

The UHPLC fingerprints of various *L*. *gracile* were established in this investigation, and 21 common peaks were chosen. DPPH, ABTS, and FRAP tests were used to assess the antioxidant properties of several *L*. *gracile* samples. The antioxidant chemicals were then discovered by looking at the spectrum–effect relationship between UHPLC fingerprints and their antioxidant activities. The Pearson correlation analysis and GRA results showed that 11 compounds that were identified as 6 flavonoids and 5 phenolic acids contributed most to the antioxidant effects of *L*. *gracile* and were selected as potential quality markers. The established spectrum–effect relationship‐based approach is of great significance for elucidating the pharmacodynamic material basis, screening core quality markers related to medicinal effect, and ensuring the safety and rational application of traditional herbal medicines.

## CONFLICT OF INTEREST

The authors declare that there is no conflict of interest.

## Supporting information

Supplementary Material

## Data Availability

Data available on request from the authors.
