# Supplementary material for: Spectrum–effect relationship between ultra‐high‐performance liquid chromatography fingerprints and antioxidant activities of Lophatherum gracile Brongn
Source: Food Sci Nutr. 2022 Feb 22;10(5):1592–601. doi: 10.1002/fsn3.2782 (PMC9094454; doi:10.1002/fsn3.2782)
Supplement: Supplementary file 1 — Supplementary Material [file FSN3-10-1592-s001.docx]

**Table S1** The information of *L. gracile* samples.

| **No.** | **source** | **Collection Time** |
| --- | --- | --- |
| **S1** | Bozhou City, Anhui Province | 2018.08 |
| **S2** | Bozhou City, Anhui Province | 2018.08 |
| **S3** | Guangde Country, Anhui Province | 2018.07 |
| **S4** | Guangde Country, Anhui Province | 2018.07 |
| **S5** | Hefei City, Anhui Province | 2018.09 |
| **S6** | Dazhou City, Sichuan Province | 2018.08 |
| **S7** | Dazhou City, Sichuan Province | 2018.08 |
| **S8** | Dazhu Country, Dazhou City, Sichuan Province | 2018.08 |
| **S9** | Dazhu Country, Dazhou City, Sichuan Province | 2018.08 |
| **S10** | Kaijiang Country, Dazhou City, Sichuan Province | 2018.08 |
| **S11** | Dujiangyan Country, Chengdu City, Sichuan Province | 2017.08 |
| **S12** | Dujiangyan Country, Chengdu City, Sichuan Province | 2017.08 |
| **S13** | Chengdu City, Sichuan Province | 2017.07 |
| **S14** | Chengdu City, Sichuan Province | 2017.07 |
| **S15** | Chengdu City, Sichuan Province | 2017.07 |


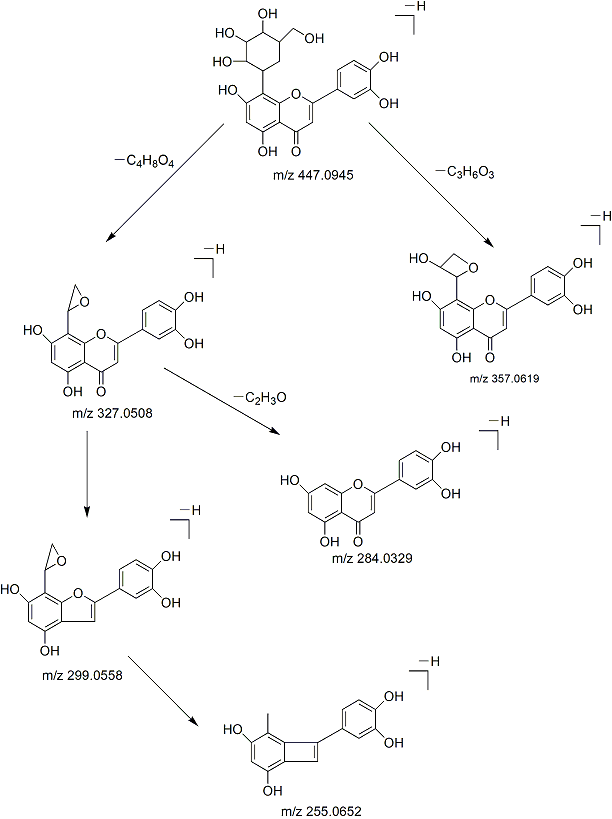


**Figure S1** Proposed fragmentation pathways of isoorientin in negative ion mode.


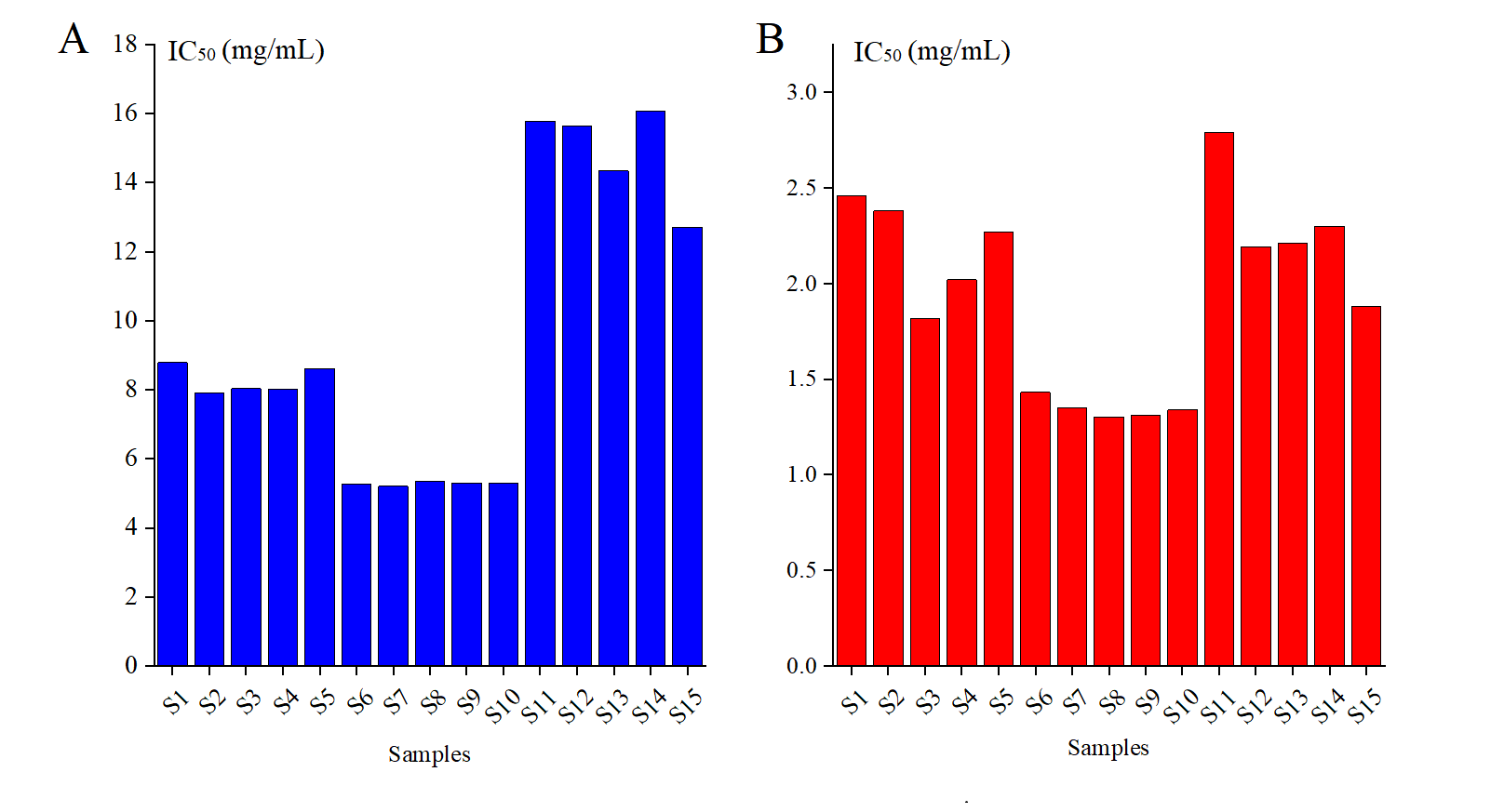


**Figure S2** The IC_50_ results of DPPH (A) and ABTS (B) antioxidant assay.
